# Chloroform a Test of Simulated Disease

**Published:** 1848-05

**Authors:** 


*Chloroform a test of Simulated Disease.*—When ether occupied the same prominent position among medical topics as now does chloroform, we directed our readers' attention to the applicability of the former to the detection of simulated disease of various kinds, as illustrated by cases occurring to the army medical officers of France and England. As might, *a priori*, be anticipated, chloroform may be used with a like intent. Several instances of its use in this way are on record in the foreign journals; but we will at present confine ourselves to the conclusions at which M. Fix, a surgeon in the French army has arrived, with respect to its employment in the discrimination of real and simulated epilepsy. He says that—

1. In a true epileptic, a fit may at any time be brought on by means of chloroform.

2. In simulated epilepsy this agent produces only its usual anæsthetic and relaxing effects.

3. That ether or chloroform inhalations administered during the fit singularly augment its duration and intensity.

4. That the medico-legal application of chloroform to epilepsy may replace the long and difficult plans generally adopted.

From a series of experiments made at the Bicêtre, it has been demonstrated that by ether inhalations a fit of epilepsy may be induced at will in those liable to them, and that chloroform brings it on still more rapidly.—*Lancet.*

